# Exonuclease domain mutants of yeast *DIS3* display genome instability

**DOI:** 10.1080/19491034.2019.1578600

**Published:** 2019-02-11

**Authors:** Karissa L. Milbury, Biplab Paul, Azra Lari, Claire Fowler, Ben Montpetit, Peter C. Stirling

**Affiliations:** a Terry Fox Laboratory, British Columbia Cancer Agency, Vancouver, Canada; b Department of Cell Biology, University of Alberta, Edmonton, Canada; c Department of Viticulture and Enology, University of California, Davis, CA, USA; d Department of Medical Genetics, University of British Columbia, Vancouver, BC, Canada

**Keywords:** DIS3, exosome, genome instability, *Saccharomyces cerevisiae*, mitotic spindle, SGA

## Abstract

The exosome functions to regulate the cellular transcriptome through RNA biogenesis, surveillance, and decay. Mutations in Dis3, a catalytic subunit of the RNA exosome with separable endonuclease and exonuclease activities, are linked to multiple myeloma. Here we report that a cancer-associated *DIS3* allele, *dis3^E729K^*, provides evidence for *DIS3* functioning in mitotic fidelity in yeast. This *dis3^E729K^* allele does not induce defects in 7S→5.8S rRNA processing, although it elicits a requirement for P-body function. While it does not significantly influence cell cycle progression alone, the allele reduces the efficiency of cell cycle arrest in strains with defects in kinetochore assembly. Finally, point mutations in the exonuclease domains of yeast Dis3 elicit genome instability phenotypes; however, these *DIS3* mutations do not increase DNA damage or RNA processing defects that lead to the accumulation of polyadenylated RNA in the nucleus. These data suggest that specific *DIS3* activities support mitotic fidelity in yeast.

## Introduction

Dis3 is one of two catalytic components, along with Rrp6, of the eukaryotic RNA exosome complex. Dis3 is a 3ʹ-5ʹ exonuclease and endonuclease that is conserved from bacteria to humans, and participates in the processing and degradation of many RNA species [1] . *DIS3* mutants suffer from impaired RNA processing, defective microtubules, growth retardation, and temperature sensitivity [2–4]. Dis3 also participates in kinetochore assembly in *Schizosaccharomyces pombe* by contributing to pericentromeric chromatin silencing [4]. Most recently, studies in *Drosophila melanogaster* and *Caenorhabditis elegans* revealed that Dis3 is a target of CDK1 phosphorylation, and this phosphorylation reduces the Dis3 exonuclease function in the G2 phase of the cell cycle [5]. Having multiple enzymatic activities, contributing to almost all aspects of RNA metabolism, and showing pleiotropic phenotypes upon mutation has made the mechanisms by which Dis3 contributes to each phenotype difficult to determine.

Delineating the impact of *DIS3* perturbation has become of medical importance over the past decade as *DIS3* mutations have been identified in roughly 11% of multiple myeloma (MM) patients, particularly within the exonuclease domain [6]. MM is a genetically heterogeneous plasma cell neoplasm, responsible for 10–15% of all blood malignancies, and is characterized by activation of *MYC, KRAS, NRAS*, and *FGFR3* in addition to a host of recurrent aneuploidies, including loss of 13q14 and 17p13 [6–8]. Reduction-of-function mutations in *DIS3* seem to arise early in tumorigenesis, implicating DIS3 as a potential tumor-suppressor gene [7].

Genome instability is a hallmark of many cancers, which provides cancer cells with enhanced evolutionary capacity by increasing the potential for sequence and karyotypic changes [9]. Genome instability can be subdivided into microsatellite instability (MIN) and chromosome instability (CIN), which induce increases in mutation rate and the rate of aneuploidy, respectively [10]. CIN is generally characterized by whole chromosome gain or loss, recurrent breakage events and/or gross chromosomal rearrangements [11]. Due to the inherent molecular complexity of these events, the cellular circuits that sustain these phenotypes remain to be fully characterized. Large screens using model organisms such as *Saccharomyces cerevisiae* have allowed for the comprehensive identification of genes and pathways that when disrupted cause CIN [12,13]. Having identified a temperature sensitive (ts) allele of *DIS3* among the novel hits, the challenge is to now understand the mechanisms by which Dis3 and the many other identified factors contribute to the maintenance of genome stability.

Towards this goal, we have phenotypically characterized a yeast strain harbouring a Dis3 mutation (E729K) that is orthologous to a human mutation (E665K) first identified in a myeloma sequencing study [7]. Analysis by synthetic genetic array (SGA) identified synthetic growth defects between this *DIS3* mutant and spindle assembly checkpoint proteins and kinetochore components. The *dis3^E729K^* mutant has been studied alongside control strains that are temperature sensitive or have endonuclease or exonuclease deficiency; these strains have allowed us to specifically link Dis3 exonuclease domain function to chromosome stability, with exonuclease mutants exhibiting reduced fitness and increased CIN. Together these data link genome maintenance to the *DIS3* exonuclease domain through a mechanism likely involving the mitotic chromosome segregation apparatus.

## Results and discussion

### Characterization of DIS3 alleles

**Figure 1.**
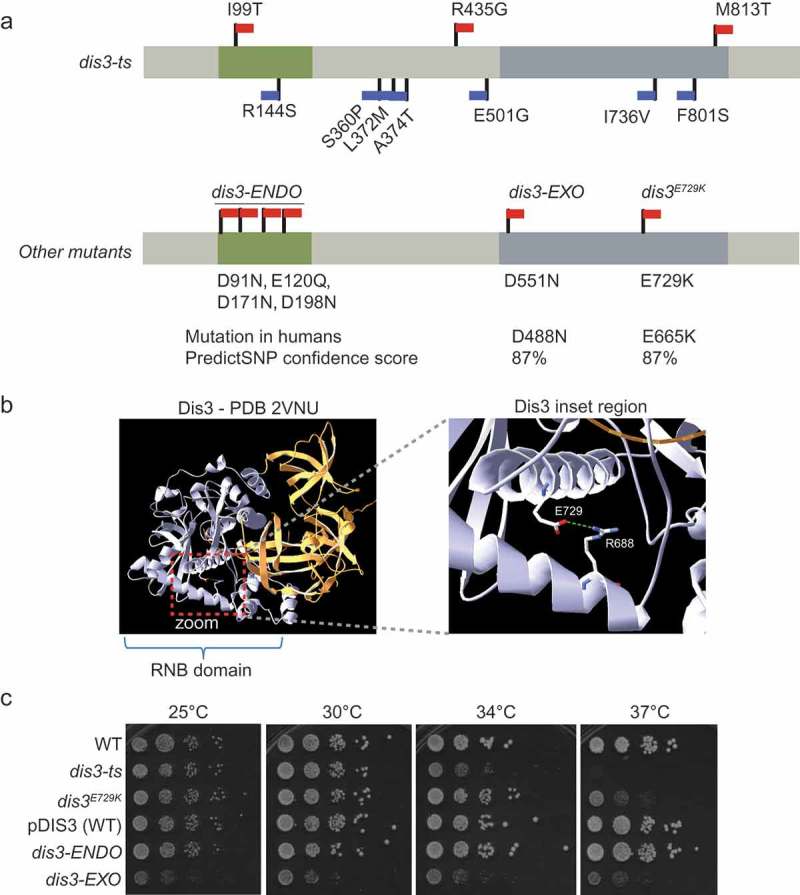
Characterization of *DIS3* separation-of-function, cancer-associated and ts-alleles. (a) Structure of major *DIS3* domains, with the non-synonymous mutations in *dis3-*ENDO, *dis3-*EXO, *dis3^E729K^*, and *dis3-*ts indicated. Red flags indicate mutations predicted to be deleterious by PredictSNP; blue flags indicate predicted neutral mutations [16]. (b) Structure of Dis3 protein highlighting the E729 residue. The RNB domain is coloured grey, while the N-terminus is coloured gold. A zoomed inset of the helices containing E729 and its hydrogen bonding partner R668 are shown on the right (a green dashed line shows the predicted H-bond) [18]. (c) Ten-fold serial dilution spots of wildtype controls versus *DIS3* mutants. Plates were incubated at the indicated temperature for 2 days prior to scanning.

Previously, a *DIS3*-temperature sensitive (ts) allele, *dis3-ts*, was found to exhibit a weak chromosome transmission fidelity (*ctf*) phenotype [14,15]. We sequenced the *dis3-ts* allele to investigate this phenotype with respect to Dis3 activity but found that *dis3-ts* carries 10 non-synonymous variants throughout the length of the gene (Figure 1(a)). As a result, it is difficult to link the *ctf* phenotype associated with this allele to any particular Dis3 domain or activity, or assess the relevance of these findings to the phenotypes of cancer cells carrying *DIS3* mutations. Thus, we engineered a disease-relevant single point mutation into budding yeast *DIS3* to investigate the influence of this mutation, in comparison to *dis3-ts*.

We chose a poorly-characterized multiple myeloma-associated point mutation, human *dis3^E665K^*, that is located within the exonuclease domain of Dis3. In yeast, *dis3^E729K^* is orthologous to the human E665K mutation and likely to be deleterious according to PredictSNP [16,17]. Analysis of a Dis3 crystal structure shows that the E729 sidechain can make a hydrogen bond contact with the guanidino moiety of R688 (Figure 1(b)) [18]. The E729K substitution would disrupt this bond, and change an acidic residue for a basic residue, which is likely to distort and/or destabilize this region of the RNB domain. In addition, we employed two control strains bearing mutations that inactivate either the endonuclease (*dis3-ENDO*; D91N, E120Q, D171N, and D198N), or the exonuclease (*dis3-EXO*; D551N) activity of Dis3 [19]. These strains carry the mutant *DIS3* allele on a plasmid with the genomic copy of *DIS3* deleted; for this reason, a plasmid-borne version of wildtype *DIS3* is used as an additional control (‘pDIS3-WT’). As expected, and in agreement with previous studies [2], *dis3-EXO* suffers from a growth defect at all tested temperatures, while *dis3-ENDO* appears to maintain near wildtype growth under these conditions (Figure 1(c)). Comparing the growth of these strains to *dis3-ts* and *dis3^E729K^* revealed that *dis3^E729K^* exhibits considerable temperature sensitivity (Figure 1(c)). It is notable that *dis3-EXO* also corresponds to a recurrent mutation observed in multiple myeloma [17].

### Genomic profiling of the cancer-associated dis3^e729k^ indicates mitotic defects

We wished to determine if this cancer-associated *DIS3* point mutation (i.e. *dis3^E729K^)* induced cellular defects similar to those previously reported for *dis3-ts*. An unbiased way to identify cellular pathways depending on Dis3 function is through genetic interaction profiling by synthetic genetic array (SGA) [20]. We predicted that genes in pathways that are buffering specific defects in *DIS3* mutants would appear as negative interactions in the SGA data. We profiled both the *dis3-ts* and *dis3^E729K^* alleles, as this would facilitate a direct comparison with previously reported *DIS3* SGA datasets (collected from a different *DIS3* allele). As well, we anticipated that the comparison would allow us to determine if the difference in the fitness of these mutants (i.e. temperature sensitivity at 37°C) represents a difference in the cellular effects of *dis3-ts* versus *dis3^E729K^*, or simply difference in severity of the same phenotypes.

**Figure 2.**
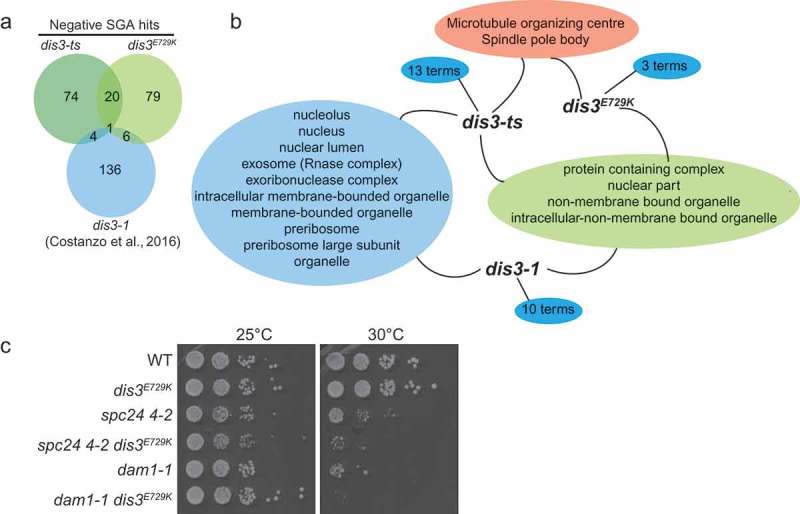
Genomic profiling of *dis3^E729K^* and *dis3-ts* indicates potential mitotic defects. (a) Overall results showing negative genetic interactions from two screens in this study, and the published *dis3-1* SGA screen [20]. (b) Analysis of gene ontology terms among three gene lists reveals specialization of DIS3 mutant allele phenotypes. (c) Spot-dilution assay validations of negative genetic interactions between *spc24* or *dam1* mutant alleles with *dis3^E729K.^*

We screened these two alleles against the yeast deletion collection comprised of non-essential gene knockouts, and collections of DAmP (Decreased Abundance by mRNA Perturbation) and ts-alleles representing >80% of all essential genes [21–23]. In total, *dis3-ts* and *dis3^E729K^* had candidate negative genetic interactions surpassing our cut-off with 94 and 99 genes, respectively (Figure 2(a), Table S1). We compared these gene lists to that published as part of a large scale study for *dis3-1* [20]. At the level of individual genes, there is little overlap in the genes identified as negative genetic interactors of *DIS3* between the tested alleles. However, at the level gene ontology (GO) cellular components, all three *DIS3* alleles elicit general genetic interactions with nuclear components, while *dis3-ts* and *dis3-1* specifically enrich for components of the exosome and those involved in ribosome biogenesis, a known function of Dis3 [24] (Figure 2(b) and Table S2). In contrast, *dis3^E729K^* and *dis3-ts* enrich specifically for components of the spindle pole body/microtubule organizing centre. The shared GO terms found between *dis3-ts* and the other two alleles suggests that the numerous point mutations in *dis3-ts* may be impact multiple Dis3 functions, while *dis3-1* and *dis3^E729K^* have interactions that are restricted to more discrete activities. We validated synthetic sick genetic interactions between *dis3^E729K^* and several SGA hits with diverse functions, including mutants involved in ribosome biogenesis (*nop2-5, bms1-1*), and mitotic mutants influencing the kinetochore (e.g. *dam1-1, spc24 4–2, mif2-3*), the Anaphase Promoting Complex (*apc2-1*), or the spindle pole body (*cdc31-2*) with spot dilution plating assays (Figure 2(c) and noted in Table S1). Together, the high-resolution phenotypes provided by these SGA screens suggests allele specificity amongst *DIS3* mutants and that *dis3^E729K^* is genetically linked to the mitotic chromosome segregation apparatus.

### DIS3 mutations induce distinct RNA processing defects

**Figure 3.**
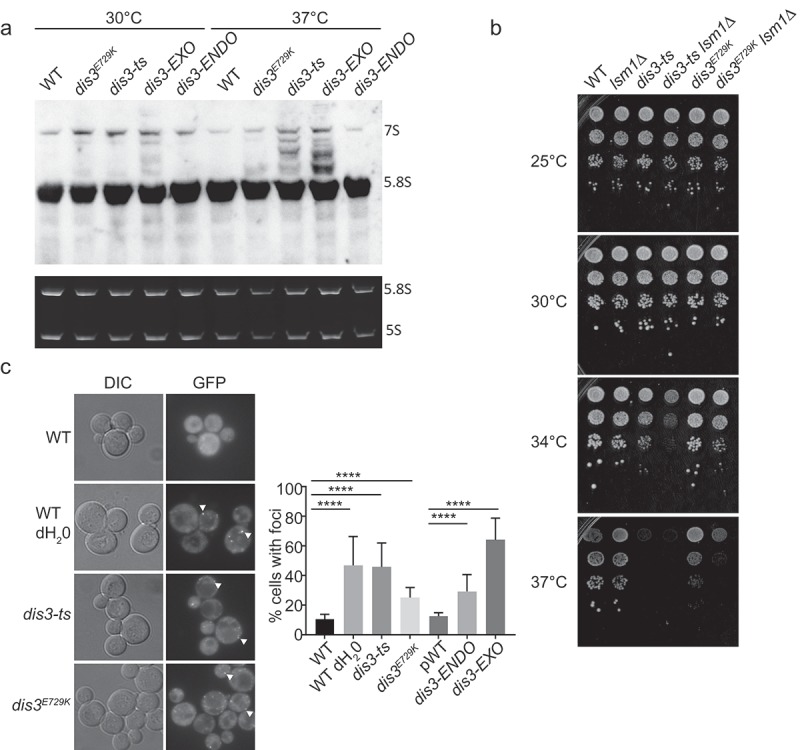
RNA processing phenotypes of *DIS3* alleles. (a) Northern blot of 5.8S rRNA maturation products in early-log phase cultures. The top panel is a 15 second exposure after hybridization with a 5.8S rRNA-specific probe, and bottom panel is an ethidium bromide stained gel demonstrating RNA loading. (b) Spot dilution assays showing genetic interaction of *dis3-ts* and *dis3^E729K^* with loss of the P-body component Lsm1. (c) Representative images (left) and quantification (right) of Lsm1-GFP labelled P-body formation in *DIS3* mutant alleles. Arrows indicate representative foci. **** indicates a p < 0.0001 via a Fisher exact test with Holm-Bonferroni correction.

Given that the SGA results for *dis3^E729K^* revealed different GO term enrichments compared with *dis3-1* or *dis3-ts*, we wished to assess if this allele would exhibit RNA processing or degradation defects that have been previously reported in *DIS3* loss-of-function mutants. We performed northern blotting to address 5.8S RNA maturation in our panel of *DIS3* mutants. As expected based on the literature and SGA screen results, we observed substantial accumulation of 5.8S processing intermediates and precursors in *dis3-ts* and *dis3-EXO* at 37°C. In contrast we observed essentially wildtype 5.8S processing in *dis3^E729K^* and *dis3-ENDO* (Figure 3(a)). This is consistent with the SGA results, as rRNA processing related terms were abundant in the *dis3-ts* (Table S2) and *dis3-1* datasets, yet were not enriched in the *dis3^E729K^* data. Notably, *dis3^E729K^* has strong growth defects at 37°C, suggesting that these growth defects arise for reasons other than changes in 5.8S rRNA processing.

Since 5.8S is just one of many transcripts engaged by Dis3, we sought a more general assay to measure cellular stress in these mutant strains. Cellular stress is known to induce requirements for parallel RNA degradation activities localized in cytoplasmic Processing-bodies (P-bodies) [25,26]. When we deleted a P-body component and mRNA 5ʹ decapping factor, *LSM1*, we found that the *dis3-ts* and *dis3^E729K^* fitness defects were greatly enhanced at 37°C, with *dis3-ts* also showing a clear growth defect at 34°C (Figure 3(b)). In support of ongoing cellular stress, all *DIS3* mutants were found to exhibit enhanced P-body formation based on Lsm1-GFP localization in the absence of exogenous stress (Figure 3(c)), but *dis3^E729K^* had the lowest levels, more similar to that of the *dis3-ENDO* allele. These data further support the idea that *dis3^E729K^* has a weaker general RNA processing defect compared to other exonuclease mutants and/or may be impacting a discrete function of Dis3. Interestingly, loss of *LSM1* has also been linked to genome instability through at least two mechanisms: one study suggested the P-bodies regulate the levels of histone mRNA during replication [27], while another study showed that P-bodies regulates the transcriptional repressor *YOX1*, leading to oxidative stress and DNA damage in *lsm1*Δ [28]. Whether these phenotypes are relevant to the observed genetic interactions with *DIS3* mutants are unknown, although the DNA damage phenotypes in *lsm1*Δ strains are not seen in our *DIS3* mutants (see below).

Finally, since temperature sensitive *dis3* alleles, and other exosome mutants, have been reported to accumulate poly(A)-RNA in the nucleolus at the non-permissive temperature [29], we asked whether this phenotype is present in *dis3^E729K^* . Fluorescence in situ hybridization (FISH) revealed that poly(A)-RNA accumulation after a 37°C temperature shift was specific to *dis3-ts* and did not occur appreciably in *dis3-ENDO, dis3-EXO*, or *dis3^E729K^* (Figure S1). Similarly, probes directed against the 5.8S rRNA and second internal transcribed spacer sequence (ITS2) region of the rRNA precursor transcript showed defects in rRNA processing in *dis3-ts* through a decrease in the nucleolar signals for both probes, which is not observed in the other mutants. Thus, while *dis3-EXO* and *dis3-ts* exhibited defects in 5.8S rRNA processing (Figure 3(a)), these changes were not accompanied by poly(A)-RNA accumulation or strong alterations in nucleolar staining with 5.8S rRNA or ITS2 probes in *dis3-EXO*. These differences again suggest that Dis3 functions within the cell are differentially perturbed at 37°C by the mutations present in the tested alleles.

### The dis3^e729k^ allele alters cell cycle progression in the context of kinetochore deficiency

**Figure 4.**
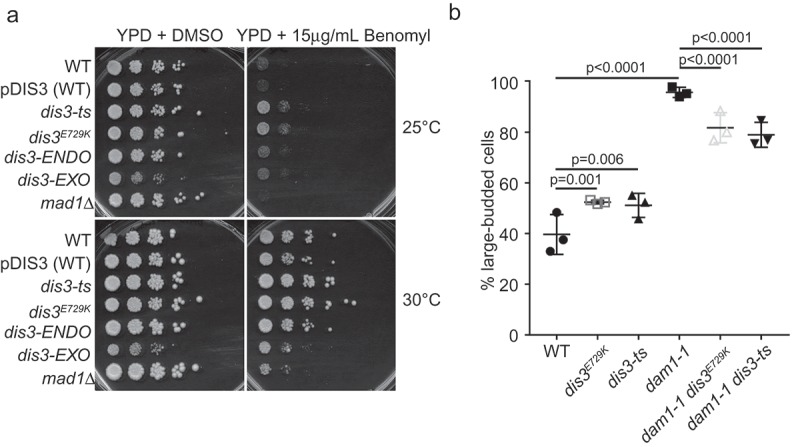
Mitosis-associated defects in *DIS3* mutants. (a) Spot dilution assays of the indicated alleles on plates with microtubule depolymerizer benomyl. (b) Budding index measurements of the indicated mutants show suppression of *dam1-1* G2/M arrest after a 3 hour temperature shift to 37°C by *dis3^E729K^* or *dis3-ts*. The Holm-Bonferroni corrected p-value determined by a Fisher Exact test is shown above each relevant comparison.

The observed phenotypic differences amongst these *DIS3* alleles in the context of our SGA analysis indicates that cell cycle functions, particularly during anaphase, are involved in the maintenance of cellular fitness in *dis3^E729K^* and *dis3-ts*. In order to assess anaphase integrity in these mutants, we plated the *DIS3* mutant strains on solid media containing microtubule poisons (benomyl and nocodazole). Both *dis3-ts* and *dis3^E729K^* showed resistance to benomyl (Figure 4(a)), and this was also evident on nocodazole plates for *dis3-ts* (Table 1). Previous work by Smith *et al*. demonstrated a benomyl and nocodazole sensitivity in *mtr17-1* [3], another *DIS3* temperature sensitivity allele, which is similar to the sensitivity seen in *dis3-EXO* at 30°C (Figure 4(a)). To further characterize this set of *DIS3* alleles, we exposed strains to a range of stresses, including DNA damaging agents (MMS), disruptors of DNA replication (hydroxyurea), a nucleotide poison (5-fluorouracil), and a topoisomerase poison (camptothecin). A summary of these results is shown in Table 1. In general, *DIS3* alleles had similar sensitivity to WT, or exhibited slight resistance to these stress treatments. Cumulatively, these data suggest that DNA damage sensitivity is not impacted by Dis3 mutations and confirms that mitotic function is likely dysregulated in *DIS3* mutants, which results in varied sensitivities to microtubule poisons.

**Table 1.** Growth behaviour of *DIS3* mutant alleles under the indicated stress.

		Fitness change (relative to wildtype)				
Temperatures tested	Condition (YPD +)	*dis3-ts*	*dis3^E729K^*	*pDIS3-WT*	*dis3-Endo*	*dis3-Exo*
25°C, 30°C, 37°C	5-FU 20 μM	0	0	0	0	0
25°C, 30°C, 37°C	MMS 0.01%	0	0	0	0	0
25°C, 30°C, 34°C, 37°C	HU 100 mM	0	0	0	0	0
25°C, 30°C, 34°C, 37°C	Camptothecin 10 μg/ml	0	0	-	-	-
25°C, 30°C, 34°C, 37°C	Nocodazole 4 μg/ml	+	0	0	+	+
25°C, 30°C, 34°C, 37°C	Benomyl 15 μg/ml	+	+	0	+	+

*(0 = no change, – = sensitive, + = resistant)

To begin to investigate the underlying cellular conditions that could be conferring the noted benomyl resistance, we first tested the budding index of *dis3-ts* and *dis3^E729K^*. These *DIS3* mutants exhibited small but significant increases in the proportion of large-budded cells suggesting a G2/M delay (Figure 4(b)). We next combined the *dis3-ts* or *dis3^E729K^* alleles with the kinetochore mutant allele *dam1-1*. While *dam1-1* alone exhibited an extreme increase of large budded cells compared to WT, indicative of a G2/M cell cycle arrest, the introduction of either the *dis3^E729K^* or *dis3-ts* allele significantly reduced the percentage of large budded cells (Figure 4(b)). This suggests that mutations in *DIS3* alter aspects of mitotic arrest arising from kinetochore dysfunction, potentially explaining the observed changes in benomyl sensitivity among *dis3* mutants, the *dis3-ts ctf* phenotype, and negative genetic interactions observed with alleles of *DIS3* and the mitotic chromosome segregation apparatus (e.g. *dam1-1 dis3^E729K^* (Figure 2(c)). Alternatively, it is also possible that *dis3-ts* and *dis3^E729K^* may slow the cell cycle and influence benomyl resistance and mitotic fidelity, as it was recently shown that slowing the yeast cell cycle can improve mitotic fidelity by allowing time for normal corrective mechanisms to work [30].

### Mutations in the DIS3 exonuclease domain cause genome instability, but not DNA damage

**Figure 5.**
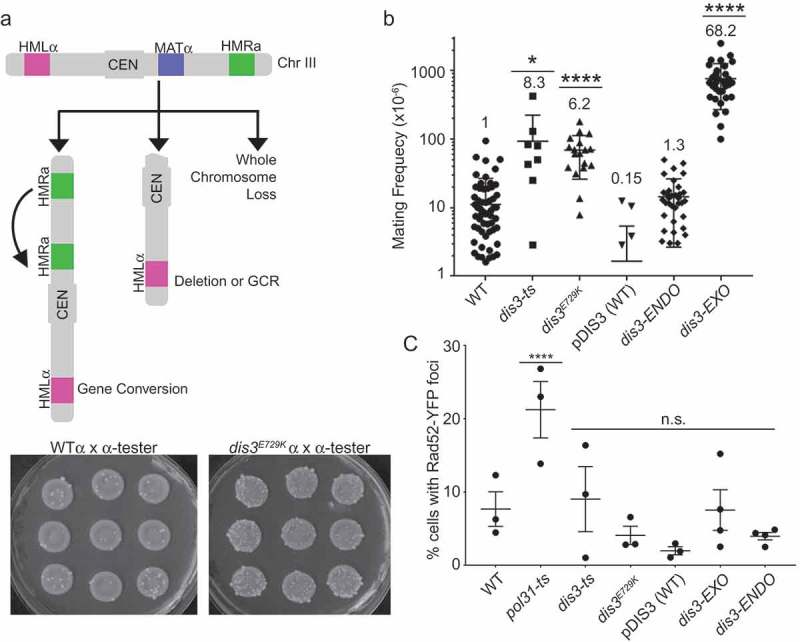
Mutations in the *DIS3* exonuclease domain cause genome instability. (a) Schematic of ALF assay. Chromosome III has active (MAT) and silent mating loci (HMR, HML). Loss of MAT by gene conversion, deletion or whole chromosome loss leads to α-α mating. Sample images of nine independent mating rate tests in WT or *dis3^E729K^* are shown below. (b) Observed frequencies of mating in ALF assay for indicated strains. The fold increase over WT is shown above each dot plot. (c) Quantification of Rad52-YFP foci formation in *DIS3* mutants. For B, data were compared with a Kruskal-Wallis Test. For C, Fisher’s Exact Test was used on raw count data. *p < 0.05, ****p < 0.0001.

Previous work showed that *dis3-ts* exhibited a CIN phenotype using an artificial chromosome fragment loss reporter [13]. Our data suggests that *dis3^E729K^* may also induce CIN, through its influence on mitotic progression in the context of kinetochore dysfunction. To test CIN on endogenous chromosomal markers, and extend this to include the *dis3^E729K^* allele, we measured instability of chr III using the A-like faker (ALF) assay that measures loss of the native MAT locus [31,32]. We observed a significant increase in the frequency of ALF colonies for both *dis3-ts* and *dis3^E729K^* (Figure 5(a,b)). We observed no increase, relative to WT for the *dis3-ENDO* strain, although the ALF rates were higher than those of the pDIS3-WT. Remarkably, when we tested ALF in the *dis3-EXO* allele we saw a dramatic 68-fold increase in mating frequency relative to WT (Figure 5(b)). While it is unclear why CIN rates are this high in *dis3-EXO*, we note that this allele completely ablates the exonuclease activity and shows slow growth at all tested temperatures, whereas *dis3-ts* and *dis3^E729K^* only show strong growth defects at temperatures above 34°C.

ALF phenotypes can reflect multiple forms of CIN, including whole chromosome loss, local deletions or chromosome arm loss, and gene conversion [31,32]. One mechanism of CIN in RNA processing mutants that could lead to an ALF phenotype is the accumulation of DNA:RNA hybrids in genomic DNA called R-loops [33]. Indeed, previous work has found that the exosome accessory complex TRAMP, and the alternative catalytic subunit Rrp6 are involved in suppressing R-loop associated CIN [34–36]. We assessed R-loop levels by staining chromosome spreads from the strains with the S9.6 antibody, which recognizes DNA:RNA hybrids [37]. While this assay confirmed the previously reported R-loop increase in *dis3-ts*, we did not find evidence for R-loops accumulating in *dis3^E729K^*. In addition, S9.6 staining did not indicate excessive R-loops accumulating in *dis3-EXO*, although we did observe a small but significant increase in this strain relative to the plasmid WT control (Figure S2). Thus, staining with S9.6 did not correlate with the CIN phenotype measured by the ALF assay. Given that R-loops typically lead to double strand breaks and hyper-recombination due to interference with DNA replication forks [38], which has been observed in other *DIS3* mutants [36,39], we also measured the frequency of Rad52-YFP foci in the *DIS3* alleles. This analysis showed no increase in Rad52 foci, further suggesting that mutant-induced DNA damage is not a likely cause of genome instability (Figure 5(c)). Indeed, this is consistent with the lack of any sensitivity to genotoxic chemicals which damage DNA (Table 1; i.e. MMS, HU, camptothecin). Consequently, these data support our hypothesis emerging from the SGA, benomyl resistance, and *dam1-1* suppression data, that mitotic spindle dysfunction could underlie the CIN phenotype of some *DIS3* alleles. Previous studies have observed mitotic apparatus defects in ts-alleles of *DIS3* [3], but did not link those defects to the exonuclease domain or to ongoing genome instability.

### Perspective

Here we characterize a new allele of *DIS3* in yeast, *dis3^E729K^*, which is orthologous to a mutation found in human cancers. Our results are consistent with this mutation, as well as *dis3-ts*, having mitotic defects, which are similar to those ascribed to the *mtr17-1* allele of *DIS3* previously shown to have abnormal mitotic spindles [3]. Our genome-wide screening data also supports this relationship with mitosis, and builds on these previous links of Dis3 to mitosis [3,4], by validating specific genetic dependencies of *DIS3* mutants (e.g. with kinetochore function) in the context of a cancer-associated point mutation that exhibits genome instability. However, hundreds of gene products have altered levels of expression in *DIS3* mutant alleles [24] and it remains difficult to confidently ascribe cellular phenotypes to specific gene expression changes. Regardless, our data suggests that exonuclease domain mutations seen in myelomas could potentially disrupt normal Dis3 functions to impact the mitotic apparatus and result in CIN.

More broadly, while various cellular pathways buffer genome stability directly (e.g. checkpoints and repair systems), genes with seemingly unrelated functions can influence DNA transactions such as repair, replication, or mitosis, if they have an impact on critical cellular processes (e.g. transcription, translation, and cellular energy status). Numerous RNA processing factors have now been implicated in genome maintenance and there are at least two possible models [40]. One model invokes the accumulation of unprocessed RNA leading to R-loops (DNA:RNA hybrids), which impair DNA replication causing DNA damage. The other model involves changes in gene expression that dysregulate more direct genome maintenance pathways such as DNA repair or mitotic function. In mutants of the *DIS3* exonuclease domain, we do not find evidence of R-loops or DNA damage, suggesting that altered expression of genome maintenance factors is more likely driving genome instability. However, we cannot rule out a more direct role for Dis3 in mitosis. Regardless, the consequences of mitotic dysregulation by *DIS3* mutants lead us to a speculative model in which specific myeloma-associated mutations impair normal cellular mitotic functions. In the longer term, through understanding the impact of cancer specific Dis3 mutations on molecular function, we expect that this information can be applied to a more complete description of *DIS3* mutant function in myeloma.

## Materials and methods

### Yeast growth and strains

Yeast were grown in rich media at 30°C by standard procedures unless otherwise noted. The pDIS3-WT, *dis3-EXO* and *dis3-ENDO* alleles were created by plasmid shuffle [19]. Additional strains were taken from the collections described below (see Methods: ‘synthetic genetic array’). Strains used in this study are listed in Table S3.

### Chromosome instability assays

For A-like faker (ALF), 9 independent colonies of each *MATα* mutant strain were inoculated overnight at permissive temperature in complete media. The next day, 100 μl of the saturated overnight cultures was mixed with 300 μL of a *MATα* mating tester strain, spun down, and resuspended in 100 μl distilled water. These *MATα* mixtures were then spotted on synthetic media lacking all supplements and only permitting prototrophic mated progeny to form colonies [31]. As well, the saturated overnight cultures were diluted by 1:100,000 and plated on complete media to ascertain colony viability. Plates were incubated at 30°C, and colonies counted once visible (typically after 3–4 days). Frequencies were calculated by dividing the number of colonies on depleted media by the colony count on complete media (which was multiplied by the dilution factor).

### Synthetic genetic array

High-throughput screens were performed using the *MATa* non-essential gene deletion collection [21], a ts-allele collection [23], and the Decreased Abundance by mRNA Perturbation (DAmP) collection [22]. All array manipulation was done with a Singer RoToR HDA. Media and steps were modifed from Tong et al., 2004 [41]. Briefly, query strains were expanded on synthetic drop-out media selecting for the *DIS3* allele at permissive temperature, then mated with the collection strains on rich media for one day at 30°C. Colonies were replicated to diploid-selective media plates for one day; this selection was repeated twice. Arrayed colonies were then replicated to sporulation media and allowed to grow at 25°C for 7 days. Sporulated arrays were expanded on haploid selection media in triplicate, and allowed to grow for roughly 30 hours; this haploid selection was then repeated a second time. Array plates were then replicated to haploid selective plates that carried additional selection for the collection alleles (‘single selection’) and incubated at 30°C for 3 days. Finally, each plate was concurrently replicated to single selection, as well as double selection (which also selects for the query allele; in this case, DIS3) and allowed to grow for one further day. Output arrays were scanned as plate images and colony size was analyzed using Balony Software [42]. Candidate hits were selected with a spot size difference (control vs experimental) cut-off of −0.2 and p-value < 0.05. Positive interactions were not analyzed for the purposes of this study.

### Microscopy

Indicated strains were grown overnight at permissive temperature in appropriate drop-out selection media. The next day, the overnight cultures were diluted and imaged after 1.5 hours (once they had resumed growth), or shifted to the indicated temperatures for 2 further hours prior to imaging. Cultures were mounted on concanavalin-coated slides and imaged at 100x magnification on a fluorescent microscope (Leica) using Metamorph software (Molecular Devices). Final images were scored using Image J (rsbweb.nih.gov/ij/) as described [13,43]. For Rad52-YFP and Lsm1-GFP imaging, a minimum of 98 cells per replicate, >400 in total across all replicates were counted, imaged in DIC and YFP or GFP channels.

For chromosome spreads, log-phase cultures incubated at 30°C were spheroplasted for 45 minutes at 37°C, treated with 4% paraformaldehyde and 1% lipsol, and manually spread onto glass slides. The following day, slides were incubated for 30 minutes in blocking buffer (5% BSA, 0.2% milk in PBS) at room temperature in a humid chamber (which was also used for all subsequent incubation steps). Immunostaining was performed with S9.6 antibody (Kerafast) diluted in blocking buffer (1:1000) for 1 hour at room temperature. Slides were washed with PBS and incubated with a goat anti-mouse Cy3-conjugated secondary (Jackson ImmunoResearch 115–165-003) diluted 1:1000 in blocking buffer for 1 hour at 25°C. Slides were washed, mounted with 100 ng/ml DAPI in Vectashield mounting media (Vector Laboratories H-1000), and imaged as described above in DAPI and Cy3 channels. A minimum of 75 cells per replicate and >400 cells in total across all replicates was counted.

### Northern blot

Yeast strains were grown to early-log phase in YPD at 30°C followed by a temperature shift to 37°C for two hours. Total RNA was extracted before and after the temperature shift as described previously by Hopper, Schultz, and Shapiro [44]. Briefly, cell pellets were re-suspended in equal volumes of ice-cold TSE buffer (0.01M Tris pH7.5, 0.01M EDTA, 0.1M sodium chloride) and TSE saturated phenol (pH = 7.4). The mixture was incubated for 20 minutes at 55°C and vortexed every 3 minutes. Phases were separated by centrifugation at 20,000g for 10 minutes and re-extracted with phenol. RNA was precipitated overnight in ethanol at −80°C.

Northern blotting was carried out as previously described [45]. 4 micrograms of total RNA was separated by electrophoresis on 10% TBE- 7M Urea gels and transferred onto a Hybond N+ membrane (Amersham). Gels were stained with 1ug/ml ethidium bromide to detect 5.8S and 5S rRNAs as loading controls. Membranes were cross-linked at 2400 J/m2 (UV Crosslinker, VWR). RNA was detected on the membrane using 10pmol/ml of a digoxigenin-labeled (DIG) probe. Probe17 sequence is 5′-GCGTTGTTCATCGATGC-3′ [46].

### Fluorescence in situ hybridization

Experiments were performed as previously described [32]. Briefly, indicated strains were grown overnight at permissive temperature in appropriate drop-out selection media. The next day, the overnight cultures were diluted and, once in log phase, shifted to 37°C for 3 hours. Cells were fixed with 5% formaldehyde for 15 min. Hybridization was performed with a FITC–labeled oligo-dT, a Cy3-labelled probe for the 5.8S rRNA and Cy5-labelled probe for the ITS2 sequence [29 #1153, 46 #1297]; cultures were then washed and mounted with mounting media/DAPI for imaging.

### Gene ontology and protein structure analysis

GO analysis was performed using the Generic GO term finder (http://go.princeton.edu/cgi-bin/GOTermFinder). Fold enrichments were calculated by computing the ratio of the number of genes associated with GO term in the hitlist and the number of genes associated with the GO term in the genome. The Dis3 crystal structure (PDB: 2VNU) [18] was visualized in DeepView (http://www.expasy.org/spdbv/) [47].

## References

[1] Robinson SR, Oliver AW, Chevassut TJ, et al The 3ʹ to 5ʹ exoribonuclease DIS3: from structure and mechanisms to biological functions and role in human disease. Biomolecules. 2015;5:1515–1539. 26193331 10.3390/biom5031515 PMC4598762

[2] Tomecki R, Drazkowska K, Kucinski I, et al Multiple myeloma-associated hDIS3 mutations cause perturbations in cellular RNA metabolism and suggest hDIS3 PIN domain as a potential drug target. Nucleic Acids Res. 2014;42:1270–1290. 24150935 10.1093/nar/gkt930 PMC3902924

[3] Smith SB, Kiss DL, Turk E, et al Pronounced and extensive microtubule defects in a Saccharomyces cerevisiae DIS3 mutant. Yeast. 2011;28:755–769. 21919057 10.1002/yea.1899 PMC3367412

[4] Murakami H, Goto DB, Toda T, et al Ribonuclease activity of Dis3 is required for mitotic progression and provides a possible link between heterochromatin and kinetochore function. PLoS One. 2007;2:e317. 17380189 10.1371/journal.pone.0000317 PMC1820850

[5] Snee MJ, Wilson WC, Zhu Y, et al Collaborative control of cell cycle progression by the RNA exonuclease DIS3 and RAS is conserved across species. Genetics. 2016;203:749–762. 27029730 10.1534/genetics.116.187930 PMC4896191

[6] Ryu D, Kim HJ, Joung JG, et al Comprehensive genomic profiling of IgM multiple myeloma identifies IRF4 as a prognostic marker. Oncotarget. 2016;7:47127–47133. 27223072 10.18632/oncotarget.9478 PMC5216929

[7] Weissbach S, Langer C, Puppe B, et al The molecular spectrum and clinical impact of DIS3 mutations in multiple myeloma. Br J Haematol. 2014 DOI:10.1111/bjh.13256. 25521164

[8] Chapman MA, Lawrence MS, Keats JJ, et al Initial genome sequencing and analysis of multiple myeloma. Nature. 2011;471:467–472. 21430775 10.1038/nature09837 PMC3560292

[9] Hanahan D, Weinberg RA. Hallmarks of cancer: the next generation. Cell. 2011;144:646–674. 21376230 10.1016/j.cell.2011.02.013

[10] Geigl JB, Obenauf AC, Schwarzbraun T, et al Defining ‘chromosomal instability’. Trends Genet. 2008;24:64–69. 18192061 10.1016/j.tig.2007.11.006

[11] Pfau SJ, Amon A Chromosomal instability and aneuploidy in cancer: from yeast to man. EMBO Rep. 2012;13:515–527. 22614003 10.1038/embor.2012.65 PMC3367249

[12] Stirling PC, Shen Y, Corbett R, et al Genome destabilizing mutator alleles drive specific mutational trajectories in Saccharomyces cerevisiae. Genetics. 2014;196:403–412. 24336748 10.1534/genetics.113.159806 PMC3914614

[13] Stirling PC, Bloom MS, Solanki-Patil T, et al The complete spectrum of yeast chromosome instability genes identifies candidate CIN cancer genes and functional roles for ASTRA complex components. PLoS Genet. 2011;7:e1002057. 21552543 10.1371/journal.pgen.1002057 PMC3084213

[14] Stirling PC, Crisp MJ, Basrai MA, et al Mutability and mutational spectrum of chromosome transmission fidelity genes. Chromosoma. 2012;121:263–275. 22198145 10.1007/s00412-011-0356-3 PMC3350768

[15] Ben-Aroya S, Coombes C, Kwok T, et al Toward a comprehensive temperature-sensitive mutant repository of the essential genes of Saccharomyces cerevisiae. Mol Cell. 2008;30:248–258. 18439903 10.1016/j.molcel.2008.02.021 PMC4130347

[16] Bendl J, Stourac J, Salanda O, et al PredictSNP: robust and accurate consensus classifier for prediction of disease-related mutations. PLoS Comput Biol. 2014;10:e1003440. 24453961 10.1371/journal.pcbi.1003440 PMC3894168

[17] Lionetti M, Barbieri M, Todoerti K, et al A compendium of DIS3 mutations and associated transcriptional signatures in plasma cell dyscrasias. Oncotarget. 2015;6:26129–26141. 26305418 10.18632/oncotarget.4674 PMC4694891

[18] Lorentzen E, Basquin J, Tomecki R, et al Structure of the active subunit of the yeast exosome core, Rrp44: diverse modes of substrate recruitment in the RNase II nuclease family. Mol Cell. 2008;29:717–728. 18374646 10.1016/j.molcel.2008.02.018

[19] Schneider C, Leung E, Brown J, et al The N-terminal PIN domain of the exosome subunit Rrp44 harbors endonuclease activity and tethers Rrp44 to the yeast core exosome. Nucleic Acids Res. 2009;37:1127–1140. 19129231 10.1093/nar/gkn1020 PMC2651783

[20] Costanzo M, VanderSluis B, Koch EN, et al A global genetic interaction network maps a wiring diagram of cellular function. Science. 2016;353 DOI:10.1126/science.aaf1420. PMC5661885 27708008

[21] Winzeler EA, Shoemaker DD, Astromoff A, et al Functional characterization of the S. cerevisiae genome by gene deletion and parallel analysis. Science. 1999;285:901–906. 10436161 10.1126/science.285.5429.901

[22] Breslow DK, Cameron DM, Collins SR, et al A comprehensive strategy enabling high-resolution functional analysis of the yeast genome. Nat Methods. 2008;5:711–718. 18622397 10.1038/nmeth.1234 PMC2756093

[23] Li Z, Vizeacoumar FJ, Bahr S, et al Systematic exploration of essential yeast gene function with temperature-sensitive mutants. Nat Biotechnol. 2011;29:361–367. 21441928 10.1038/nbt.1832 PMC3286520

[24] Schneider C, Kudla G, Wlotzka W, et al Transcriptome-wide analysis of exosome targets. Mol Cell. 2012;48:422–433. 23000172 10.1016/j.molcel.2012.08.013 PMC3526797

[25] Yoon JH, Choi EJ, Parker R Dcp2 phosphorylation by Ste20 modulates stress granule assembly and mRNA decay in Saccharomyces cerevisiae. J Cell Biol. 2010;189:813–827. 20513766 10.1083/jcb.200912019 PMC2878948

[26] Teixeira D, Sheth U, Valencia-Sanchez MA, et al Processing bodies require RNA for assembly and contain nontranslating mRNAs. Rna. 2005;11:371–382. 15703442 10.1261/rna.7258505 PMC1370727

[27] Herrero AB, Moreno S Lsm1 promotes genomic stability by controlling histone mRNA decay. Embo J. 2011;30:2008–2018. 21487390 10.1038/emboj.2011.117 PMC3098488

[28] Loll-Krippleber R, Brown GW P-body proteins regulate transcriptional rewiring to promote DNA replication stress resistance. Nat Commun. 2017;8:558. 28916784 10.1038/s41467-017-00632-2 PMC5601920

[29] Paul B, Montpetit B Altered RNA processing and export lead to retention of mRNAs near transcription sites and nuclear pore complexes or within the nucleolus. Mol Biol Cell. 2016;27:2742–2756. 27385342 10.1091/mbc.E16-04-0244 PMC5007094

[30] Vinton PJ, Weinert T A slowed cell cycle stabilizes the budding yeast genome. Genetics. 2017;206:811–828. 28468908 10.1534/genetics.116.197590 PMC5499188

[31] Novoa CA, Ang JS, Stirling PC The a-like faker assay for measuring yeast chromosome III stability. Methods Mol Biol. 2018;1672:1–9. 29043612 10.1007/978-1-4939-7306-4_1

[32] Yuen KW, Warren CD, Chen O, et al Systematic genome instability screens in yeast and their potential relevance to cancer. Proc Natl Acad Sci U S A. 2007;104:3925–3930. 17360454 10.1073/pnas.0610642104 PMC1820685

[33] Gomez-Gonzalez B, Garcia-Rubio M, Bermejo R, et al Genome-wide function of THO/TREX in active genes prevents R-loop-dependent replication obstacles. Embo J. 2011 DOI:10.1038/emboj.2011.206 PMC3160181 21701562

[34] Luna R, Jimeno S, Marin M, et al Interdependence between transcription and mRNP processing and export, and its impact on genetic stability. Mol Cell. 2005;18:711–722. 15949445 10.1016/j.molcel.2005.05.001

[35] Gavalda S, Gallardo M, Luna R, et al R-loop mediated transcription-associated recombination in trf4Delta mutants reveals new links between RNA surveillance and genome integrity. PLoS One. 2013;8:e65541. 23762389 10.1371/journal.pone.0065541 PMC3676323

[36] Chan YA, Aristizabal MJ, Lu PY, et al Genome-wide profiling of yeast DNA:RNA hybrid prone sites with DRIP-chip. PLoS Genet. 2014;10:e1004288. 24743342 10.1371/journal.pgen.1004288 PMC3990523

[37] Wahba L, Amon JD, Koshland D, et al RNase H and multiple RNA biogenesis factors cooperate to prevent RNA:DNA hybrids from generating genome instability. Mol Cell. 2011;44:978–988. 22195970 10.1016/j.molcel.2011.10.017 PMC3271842

[38] Chang EY, Stirling PC Replication fork protection factors controlling R-loop bypass and suppression. Genes (Basel). 2017;8 DOI:10.3390/genes8010033. PMC5295027 28098815

[39] Tsanova B, Spatrick P, Jacobson A, et al The RNA exosome affects iron response and sensitivity to oxidative stress. Rna. 2014;20:1057–1067. 24860016 10.1261/rna.043257.113 PMC4114685

[40] Chan YA, Hieter P, Stirling PC Mechanisms of genome instability induced by RNA-processing defects. Trends Genet. 2014;30:245–253. 24794811 10.1016/j.tig.2014.03.005 PMC4039741

[41] Tong AH, Lesage G, Bader GD, et al Global mapping of the yeast genetic interaction network. Science. 2004;303:808–813. 14764870 10.1126/science.1091317

[42] Young BP, Loewen CJ Balony: a software package for analysis of data generated by synthetic genetic array experiments. BMC Bioinformatics. 2013;14:354. 24305553 10.1186/1471-2105-14-354 PMC4234492

[43] Schneider CA, Rasband WS, Eliceiri KW NIH Image to ImageJ: 25 years of image analysis. Nat Methods. 2012;9:671–675. 22930834 10.1038/nmeth.2089 PMC5554542

[44] Hopper AK, Schultz LD, Shapiro RA Processing of intervening sequences: a new yeast mutant which fails to excise intervening sequences from precursor tRNAs. Cell. 1980;19:741–751. 7363329 10.1016/s0092-8674(80)80050-x

[45] Wu J, Bao A, Chatterjee K, et al Genome-wide screen uncovers novel pathways for tRNA processing and nuclear-cytoplasmic dynamics. Genes Dev. 2015;29:2633–2644. 26680305 10.1101/gad.269803.115 PMC4699390

[46] Allmang C, Mitchell P, Petfalski E, et al Degradation of ribosomal RNA precursors by the exosome. Nucleic Acids Res. 2000;28:1684–1691. 10734186 10.1093/nar/28.8.1684 PMC102825

[47] Guex N, Peitsch MC SWISS-MODEL and the Swiss-PdbViewer: an environment for comparative protein modeling. Electrophoresis. 1997;18:2714–2723. 9504803 10.1002/elps.1150181505

