# Supplementary material for: Exonuclease domain mutants of yeast DIS3 display genome instability
Source: Nucleus. 2019 Feb 11;10(1):21–32. doi: 10.1080/19491034.2019.1578600 (PMC6380420; doi:10.1080/19491034.2019.1578600)
Supplement: Supplemental Material [file kncl-10-01-1578600-s001.zip › Supplementary information/Supplementary Information List.docx]

**Supplementary Information List**

**Figure S1**. Representative images of polyA RNA FISH experiments in the indicated *DIS3* mutant strains. Samples are co-stained by FISH for poly(A)-mRNA, RDN5.8S, the ITS2 spacer, and total DNA with DAPI. Scale bar (lower right) indicates 2μm.

**Figure S2**. Detection of R-loop staining in a panel of *DIS3* mutant alleles. Immunofluorescent detection of R-loop accumulation by S9.6 on chromosome spreads. Nuclear material was stained with DAPI, and S9.6 detected using a Cy3-conjugated secondary antibody. Error bars indicate Standard Error of the Mean. Fisher Exact Test on pooled count data for three replicates with Bonferroni multiple hypothesis correction was used to calculate statistical significance.

**Table S1.**  SGA hit lists for *dis3-ts* and *dis3^E729K^*

**Table S2**. Gene Ontology analysis of high-throughput data

**Table S3**. Yeast strains used in this study.
