# Supplementary figures and images for: Exonuclease domain mutants of yeast DIS3 display genome instability
Source: Nucleus. 2019 Feb 11;10(1):21–32. doi: 10.1080/19491034.2019.1578600 (PMC6380420; doi:10.1080/19491034.2019.1578600)

A

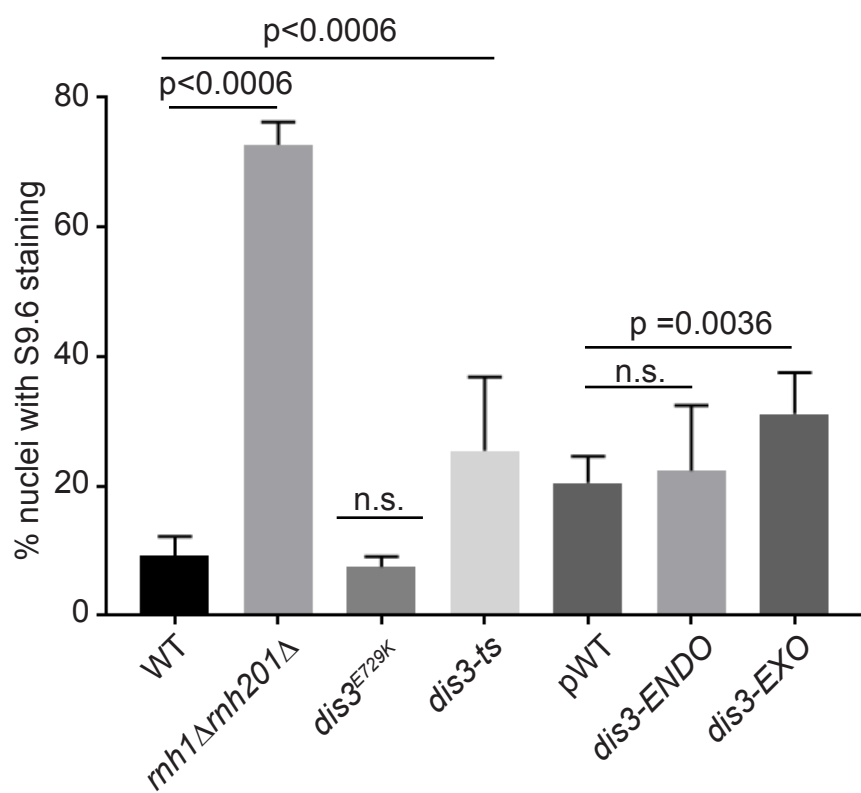

Figure-S2 (Milbury)

Supplement: Supplemental Material [file kncl-10-01-1578600-s001.zip › Supplementary information/Figure S2_Revised.pdf]

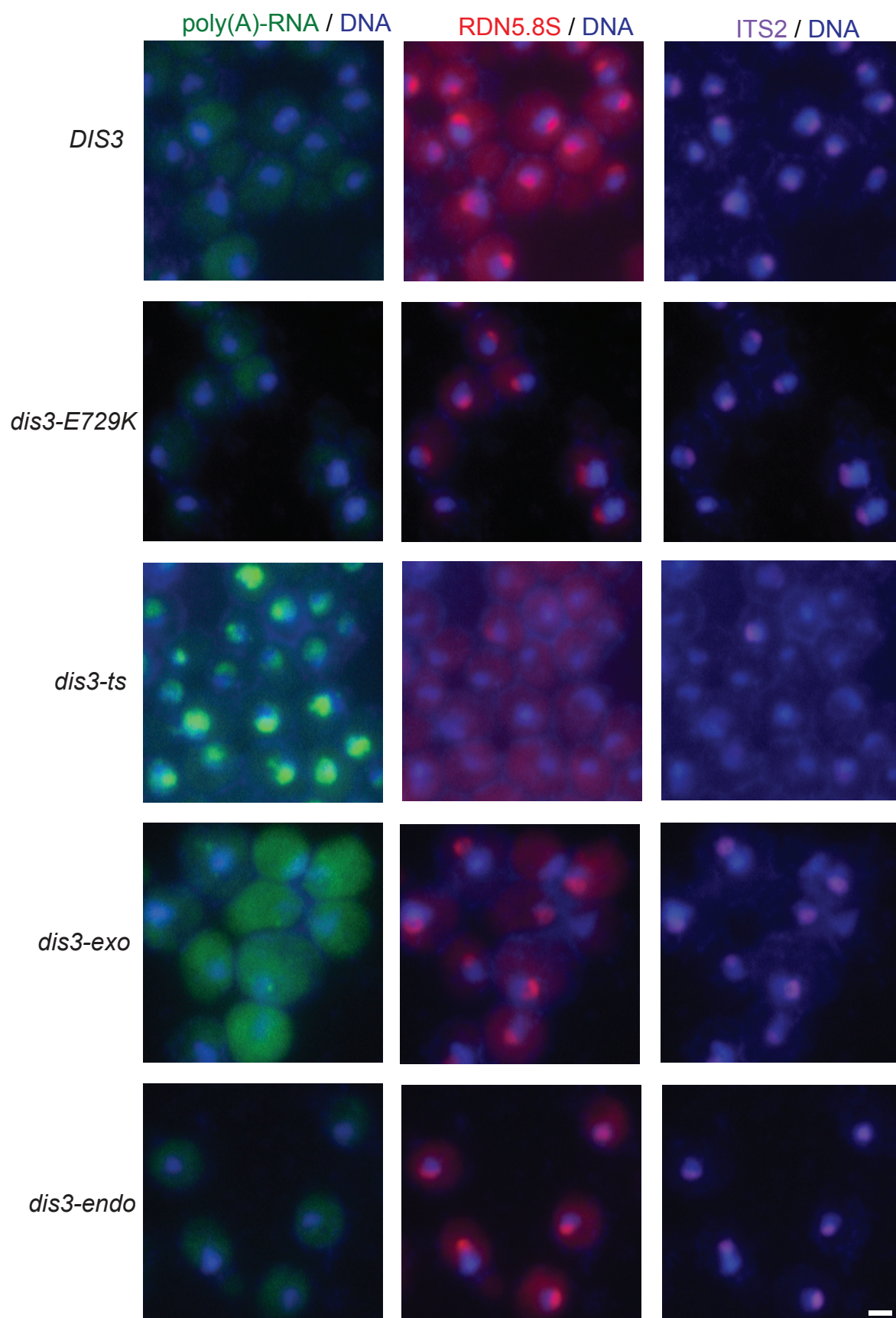

Figure-S1 (Milbury)

Supplement: Supplemental Material [file kncl-10-01-1578600-s001.zip › Supplementary information/FigureS1_Revised.pdf]
